# Salinity Independent Flow Measurement of Vertical Upward Gas-Liquid Flows in a Small Pipe Using Conductance Method

**DOI:** 10.3390/s20185263

**Published:** 2020-09-15

**Authors:** Dayang Wang, Ningde Jin, Lusheng Zhai, Yingyu Ren

**Affiliations:** 1 School of Electrical and Information Engineering, Tianjin University, Tianjin 300072, China; wangdayang@tju.edu.cn (D.W.); lszhai@tju.edu.cn (L.Z.); renyingyuee@tju.edu.cn (Y.R.); 2 College of Information Science and Engineering, Northeastern University, Shenyang 110819, China

**Keywords:** gas-liquid two-phase flow, conductance method, water holdup measurement model, flow velocity measurement models, salinity independent flow measurement

## Abstract

Flow measurement in gas-liquid two-phase flow is always a challenging work, because of the non-uniform phase distribution, severe slippage effect between phases, and different flow structures. Furthermore, the variation of salinity changes the water conductivity, which brings more difficulties to multiphase flow measurement. In this study, a methodology for flow measurement using the conductance method in gas-liquid two-phase flow with salinity change is proposed. The methodology includes the suitable conductivity detection method, the strategy of using combined sensors, and the measurement models of flow parameters. A suitable conductivity detection method that can guarantee that the sensor output is linearly proportional to the conductivity is proposed. This conductivity detection method can ensure that the sensors have a high and constant resolution in the conductivity variation caused by water holdup under the conditions of water conductivity change. Afterward, a combined sensor system consisting of a water holdup sensor, velocity sensor, and water conductivity sensor is designed and experimentally evaluated in gas-water two-phase flow in a 20 mm inner diameter pipe. Considering the non-uniform phase distribution, severe slippage effect between phases, different flow structures, and the variation of salinity, a new water holdup measurement model and flow velocity measurement models are established to achieve salinity independent water holdup measurement and flow velocity measurement for the first time.

## 1. Introduction

Gas-liquid two-phase flow is common in the gas producing well of oil fields. Accurate knowledge about the water holdup and flow velocity is greatly significant for the optimization of oil gas field exploitation. Due to the non-uniform phase distribution, severe slippage effect between phases, and different flow structures in gas-liquid flows, the flow parameters measurement is very challenging. Moreover, the salinity of the produced water usually varies in the well because of the different salinity of the formation water in different areas and the salinity change during the oil well production. Variable salinity changes the water conductivity, which brings more difficulty to multiphase flow measurement.

Several studies have focused on the measurement of water holdup with varying salinity. The gamma radiation technology [1,2] has been investigated to achieve salinity independent water holdup measurement. Strict safety protection should be carried out because of the radioactivity. The microwave technology [3,4,5,6] also performs well in water conductivity measurement and water holdup measurement, which usually requires a relatively complex measurement system. The electrical methods, which mainly include the conductance method [7,8,9,10,11,12,13,14,15,16] and capacitance method [17,18,19], have been widely applied in multiphase flow measurement since they have the advantages of high reliability, fast response, and relative simplicity of operation. For the water continuous multiphase flow, the conductance method is superior to the capacitance method. However, the responses of conductance sensors are seriously affected by the water conductivity change. In horizontal oil-water flows with varying salinity, researchers have proposed a novel conductance sensor to measure the water conductivity, which can realize the calibration of salinity in water holdup measurement [16]. For the vertical upward gas-water flows with a wide range of water conductivity in this study, in order to achieve water holdup measurement using the conductance method, a suitable conductivity detection method needs to be investigated. The suitable conductivity detection method should possess a high and constant resolution in the conductivity variation caused by water holdup under the conditions of water conductivity change. Moreover, the method of eliminating the effect of salinity on the water holdup measurement, and the establishment of a valid water holdup measurement model urgently needs to be investigated. For the flow velocity measurement, the cross-correlation method is widely applied to the measurement of mixture velocity [20,21,22,23,24,25]. However, the performance of a cross-correlation flowmeter using the conductance method under the conditions of water conductivity change has seldom been investigated. In addition, due to the severe slippage effect between phases, and non-uniform phase distribution, valid flow velocity measurement models need to be investigated to achieve mixture velocity and individual phase superficial velocity based on the measured water holdup and cross-correlation velocity.

In this study, a methodology is proposed to achieve flow measurement in vertical upward gas-liquid two-phase flow, which presents the characteristics of a non-uniform phase distribution, severe slippage effect between phases, different flow structures, and the variable salinity. We firstly investigated the conductivity detection method, which is suitable for the condition of water conductivity variation. Then, a strategy of using combined sensors to realize the flow measurement is proposed, and a combined sensor system was designed and experimental evaluated. Considering the characteristics of the gas-water flows, the measurement models, including the water holdup measurement model, the mixture velocity measurement model, and individual phase superficial velocity measurement model, were established to achieve salinity independent flow measurement in gas-water flows using the conductance method for the first time.

## 2. Conductivity Detection Method

**Figure 1:**
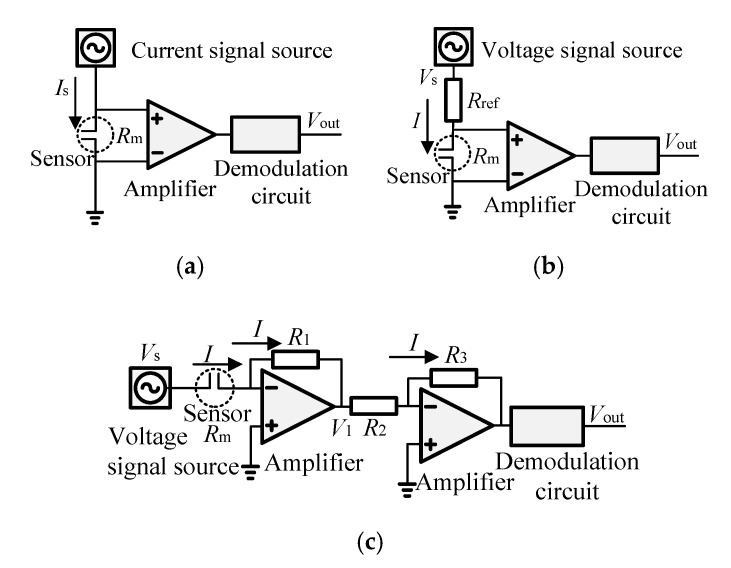
The circuits of three typical conductivity detection methods: (**a**) Method one; (**b**) Method two; (**c**) Method three.

Three conductivity detection methods can be designed to obtain conductivity. As shown in Figure 1a, method one uses a constant current as the exciting signal. Supposing the value of the exciting current signal, the equivalent resistance, and equivalent conductance between the two electrodes of sensor are *I*_s_, *R*_m_, and *G*, respectively, therefore:

(1) $$V_{\mathrm{out}}=NI_{s}R_{m}$$

where *N* is the amplification factor. As $R_{m}=1/G=1/k\sigma$, then:

(2) $$V_{\mathrm{out}}=NI_{s}/G=NI_{s}/k\sigma =a_{1}/\sigma$$

where $a_{1}=NI_{s}/k$, *k* is a constant relating to the configuration of the electrode, and σ is the conductivity.

As shown in Figure 1b, method two applies a constant voltage signal to excite the exciting electrode through a reference resistant *R*_ref_ and demodulates the voltage on the sensor to acquire the conductivity. The sensor output is:

(3) $$V_{\mathrm{out}}=NIR_{m}=NV_{s}R_{m}/(R_{m}+R_{\mathrm{ref}})$$

where *V*_s_ is the value of the exciting voltage signal. Since $R_{m}=1/G=1/k\sigma$, then:

(4) $$V_{\mathrm{out}}=NV_{s}/(1+R_{\mathrm{ref}}k\sigma )=a_{2}/(1+b_{2}\sigma )$$

where $a_{2}=NV_{s}$, and $b_{2}=R_{\mathrm{ref}}k$.

For the purpose of obtaining the positive sensor output, which is linear with the conductivity, we designed the circuit of method three, which uses a constant voltage as the exciting signal, and demodulates the current in the sensor to obtain the conductivity as shown in Figure 1c. The current in *R*_m_ is equal to the current in *R*_1_, then:

(5) $$V_{s}/R_{m}=-V_{1}/R_{1}$$

Considering $R_{m}=1/G=1/k\sigma$, then:

(6) $$V_{1}=-V_{s}R_{1}G=-V_{s}R_{1}k\sigma$$

Because of the inverting amplifier, the relationship between *V*_1_ and *V*_out_ is:

(7) $$V_{1}/R_{2}=-V_{\mathrm{out}}/R_{3}$$

The sensor output can be expressed as:

(8) $$V_{\mathrm{out}}=V_{s}R_{1}R_{3}k\sigma /R_{2}=a_{3}\sigma$$

where $a_{3}=V_{s}R_{1}R_{3}k/R_{2}$.

**Figure 2:**
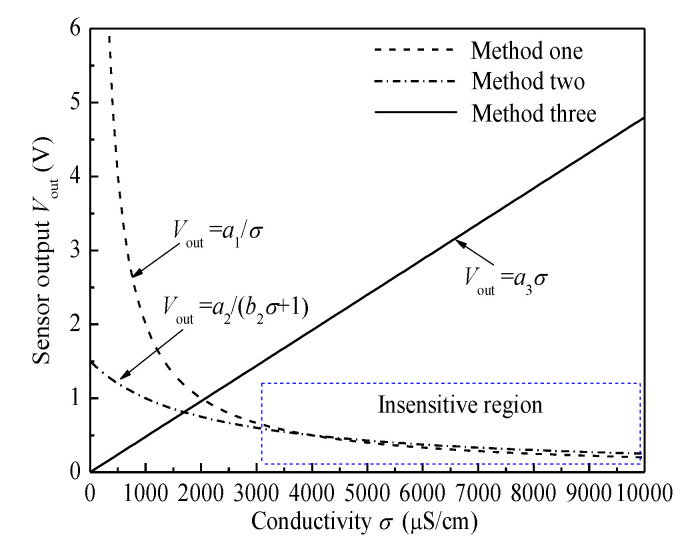
The relationships between the conductivity and the sensor output of three typical conductivity detection methods.

Although the values of *a*_1_, *a*_2_, *b*_2_, and *a*_3_ will vary with the configuration of electrodes and the circuit parameters, the relationships between the conductivity and the sensor output in each method obey the same function form. Therefore, we give the relationships between the conductivity and the sensor output in each method as shown in Figure 2. It can be seen that, for method one and method two, with the increase of conductivity, the resolution of the sensor to the change of conductivity decreases sharply, and there is an insensitive region to the change of conductivity. When the conductivity of the solution is low, such as under the condition of tap water, these two methods have good resolution and are widely used. However, when the conductivity of water varies in a large range, the sensor response will be in an insensitive region, and the resolution of the sensor will be reduced. This low resolution limits their application in extracting the small conductivity fluctuation caused by the change of water holdup in high water conductivity conditions. For the improved conductivity detection method (method three), the sensor output is positively linearly proportional to the conductivity. The resolution of the sensor to the conductivity change is not affected by the large range change of the conductivity. Therefore, method three, which can obtain a linear relationship between the sensor output and the conductivity, is more suitable for detecting conductivity variation caused by the change of water holdup under the conditions of water conductivity change with a wide range in multiphase flows.

## 3. The Strategy of Using Combined Sensors

**Figure 3:**
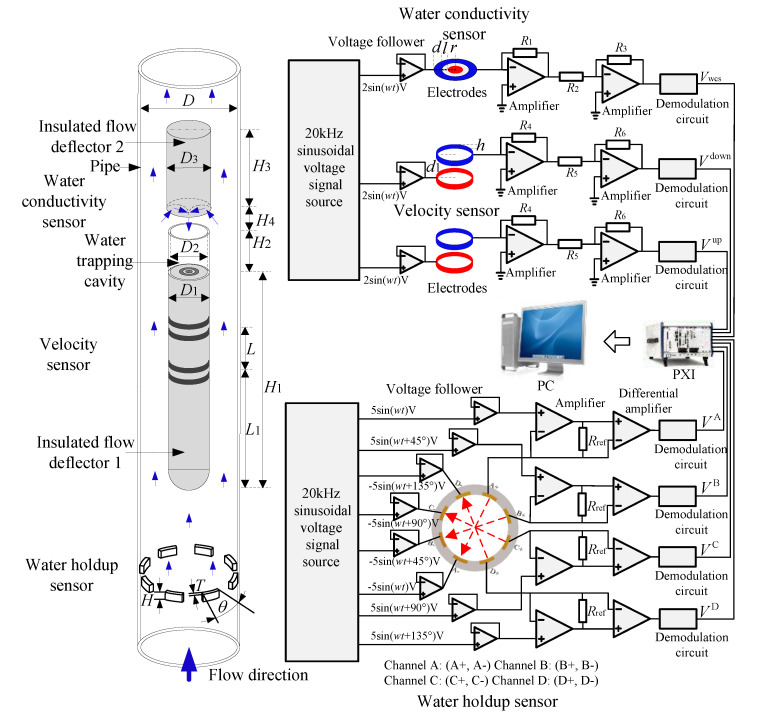
The combined conductance sensor system.

For the gas-liquid two-phase flow, the water holdup and the mixture velocity are the two main measurement parameters. Based on these two parameters, the individual superficial velocity can be derived. For the water holdup measurement, the water holdup sensor must have a sensitive and homogenous detection field to deal with the non-uniform phase distribution and different flow structures. Due to the variable water conductivity, a water conductivity sensor is needed to acquire the water conductivity information. For the mixture velocity measurement, the velocity sensor must be able to acquire the cross-correlation velocity and make it easy to deduce the mixture velocity from the cross-correlation velocity. Based on this strategy mentioned above, we designed a combined sensor system, which is shown in Figure 3. The rotating electric field conductance sensor, which has a sensitive and homogenous detection field, was selected as the water holdup sensor. The water holdup sensor consists of four pairs of electrodes, and the field angle *θ*, axial height *H*, and radial thickness *T* of the electrodes are 22.5°, 4 mm, and 1 mm, respectively [26]. In this study, in order to apply it to the gas-water two-phase flow with water conductivity change, its circuit was improved. The eight-channel sinusoidal voltage signals with 20 kHz frequency and phase difference of 45° are generated by a designed sinusoidal voltage signal source. Because of the salinity change in gas-water two-phase flow, the voltage signal source must have a very high load capacity. Therefore, in this study, eight-channel sinusoidal voltage signals were applied to the electrodes through eight voltage followers, which can improve the load capacity. In addition, the circuit parameters were designed to make the circuit work well under the water conductivity variation conditions. The outputs of the four channels $V_{}^{A}$, $V_{}^{B}$, $V_{}^{C}$, and $V_{}^{D}$, are:

(9) $$\left\{ \begin{array}{ll} V_{}^{A}=NR_{\mathrm{ref}}(2V_{s})k_{}\sigma_{}^{A} & \mathrm{Channel}\;A\\ V_{}^{B}=NR_{\mathrm{ref}}(2V_{s})k_{}\sigma_{}^{B} & \mathrm{Channel}\;B\\ V_{}^{C}=NR_{\mathrm{ref}}(2V_{s})k_{}\sigma_{}^{C} & \mathrm{Channel}\;C\\ V_{}^{D}=NR_{\mathrm{ref}}(2V_{s})k_{}\sigma_{}^{D} & \mathrm{Channel}\;D \end{array}\right.$$

where *N* is the amplification factor, and *V*_s_ is the value of exciting voltage signals. The constant *k* is related to the configuration of each pair of electrodes, and $\sigma_{}^{A}$, $\sigma_{}^{B}$, $\sigma_{}^{C}$, and $\sigma_{}^{D}$ are the conductivities measured from four channels. As $NR_{\mathrm{ref}}(2V_{s})k_{}$ is constant, the sensor outputs are linearly proportional to the conductivities.

The configuration of the velocity sensor combined with the water conductivity sensor is shown in Figure 3. A center body (insulated flow deflector 1) with a length *H*_1_ = 200 mm, and diameter *D*_1_ = 10 mm is fixed in the middle of the pipe, and upstream and downstream conductance sensors with ring-shaped electrodes are mounted on the insulated flow deflector 1. The distance *L* between two sensors is 30 mm and the distance *L*_1_ between the upstream sensor and the head of the insulated flow deflector 1 is 100 mm. For each conductance sensor, the height *h* of the electrode and the distance between two ring-shaped electrodes *d*_1_ is 2 and 7 mm, respectively [27]. In this study, at the tail of the center body, a water trapping cavity was designed to form the water region. The electrodes of water conductivity sensor is installed in it to acquire the water conductivity. The insulated flow deflector 2 is used to lead the new water into the water trapping cavity, which can ensure that the old water can be quickly exchanged by the new water. The geometric parameters of electrodes, water trapping cavity, and insulated flow deflector 2 can be found in [28]. In order to ensure that the old water in the water trapping cavity can be quickly exchanged by new water, the distance *H*_4_ is adjusted to 2 mm according to the gas-water flow conditions. To apply these two sensors to gas-water two-phase flow with water conductivity change, we designed their circuits based on method three as mentioned above. As shown in Figure 3, a sinusoidal voltage signal source is designed to generate three sinusoidal exciting voltage signals with a 20 kHz frequency, and considering the change of water conductivity, three voltage followers were applied to improve the load capacity of the signal source in this study. Moreover, the circuit parameters were determined to make the circuit work well under the water conductivity variation conditions. According to Equation (8), the sensor output of the water conductivity sensor is:

(10) $$V_{\mathrm{wcs}}=V_{s}R_{1}R_{3}k_{}\sigma /R_{2}$$

The outputs of the velocity sensor are:

(11) $$\left\{ \begin{array}{l} V_{}^{\mathrm{up}}=V_{s}R_{4}R_{6}k_{}\sigma^{\mathrm{up}}/R_{5}\\ V_{}^{\mathrm{down}}=V_{s}R_{4}R_{6}k_{}\sigma^{\mathrm{down}}/R_{5} \end{array}\right.$$

where *V*_s_ is the value of the exciting voltage signal, and *k* depends on the configuration of different electrodes. As $V_{s}R_{1}R_{3}k_{}/R_{2}$ and $V_{s}R_{4}R_{6}k_{}/R_{5}$ are constant, the sensor outputs are linearly proportional to the conductivities.

## 4. Experimental Evaluation

**Figure 4:**
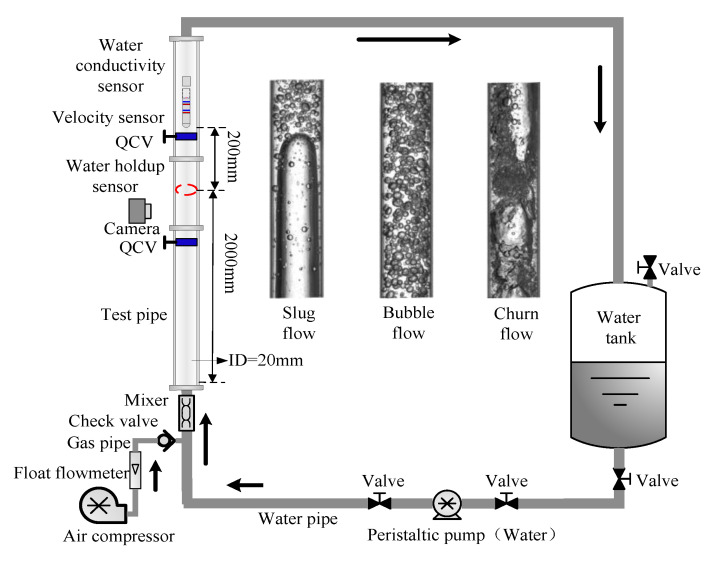
A schematic diagram of the experimental flow loop facility.

In order to verify the methodology for flow measurement in gas-water two-phase flow with water conductivity change, experiments were carried out on the flow loop facility, which is shown in Figure 4. The air produced by an air compressor and water solutions with different conductivities ($\sigma_{w}$ = 1000 μS/cm, 4000 μS/cm and 8000 μS/cm) was the experimental media. A float flowmeter metered the flux of gas, and the flux of water was transported and controlled by an industrial peristaltic pump. The test pipe with inner diameter of 20 mm was made of acrylic material, and the sensors were respectively mounted in the test pipe. In the experiment, the gas superficial velocity *U*_sg_ was set from 0.0553 to 0.5888 m/s, and the water superficial velocity *U*_sw_ ranged from 0.0368 to 1.1789 m/s. The flow patterns, including slug flow, bubble flow, and churn flow, were produced. The output signals of the sensors were all sampled by the data acquisition card PXI-4472 of NI Company. The flow patterns were recorded by a high-speed camera and the real water holdup was obtained by quick closing valves (QCVs) in the process of the experiment.

**Figure 5:**
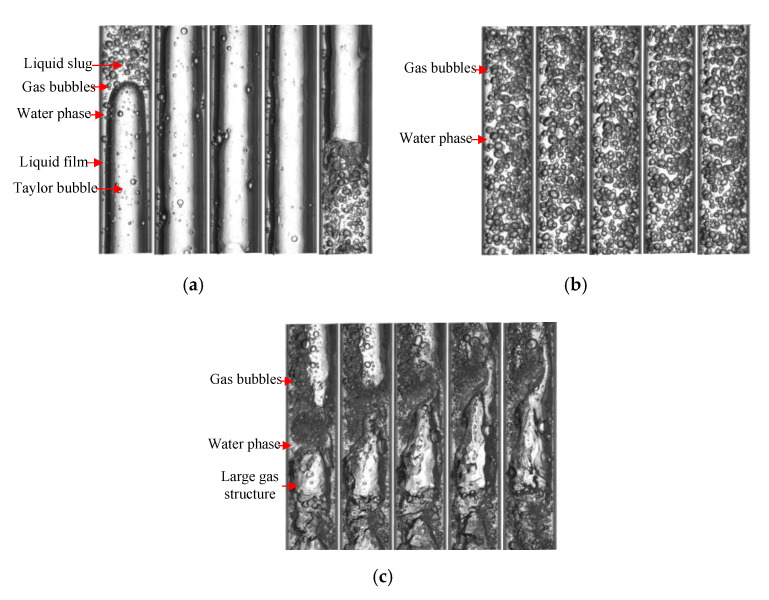
The images of three typical flow patterns: (**a**) Slug flow; (**b**) Bubble flow; (**c**) Churn flow.

The high-speed camera images of three flow patterns are shown in Figure 5, by which the flow patterns can be identified, and they can also help us understand the flow structures in the experiment. For slug flow, Taylor bubbles occupy almost the entire cross section of the pipe, and liquid film forms between the Taylor bubbles and the pipe wall. Large numbers of gas bubbles distribute in the liquid slugs, which are similar to bubble flows. Taylor bubbles and liquid slugs appear alternately. For bubble flow, large amounts of gas bubbles with a small size distribute in the water with intense random movement. In churn flow, the large gas structures are similar to Taylor bubbles, and liquid slugs are similar to bubble flows. These two structures appear to be inconsistent and disordered.

## 5. Results and Discussion

### 5.1. Salinity Independent Fluctuation Signals of Water Holdup

**Figure 6:**
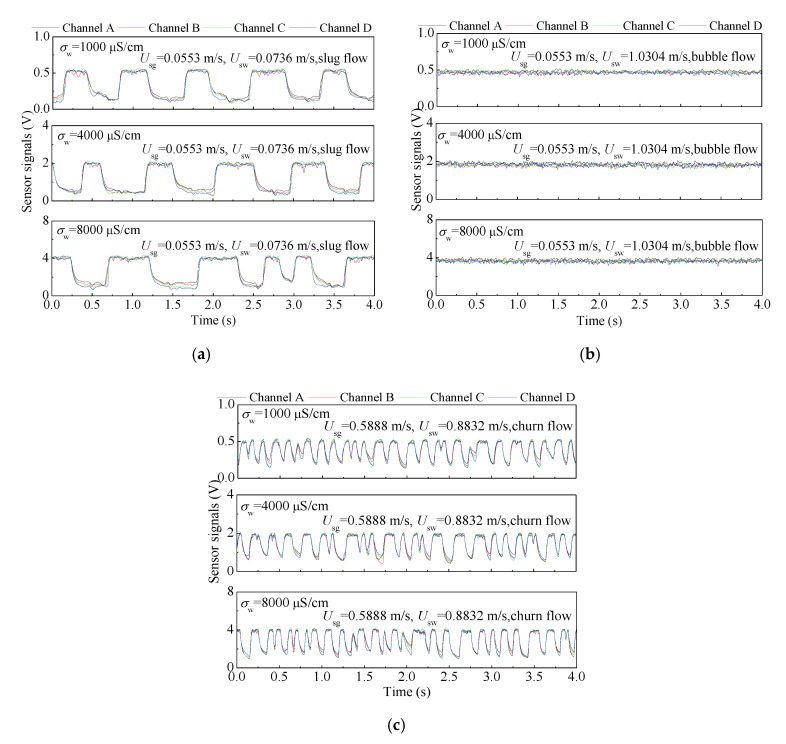
The sensor outputs of the water holdup sensor under different flow patterns with different water conductivities: (**a**) The outputs for slug flow; (**b**) The outputs for bubble flow; (**c**) The outputs for churn flow.

**Figure 7:**
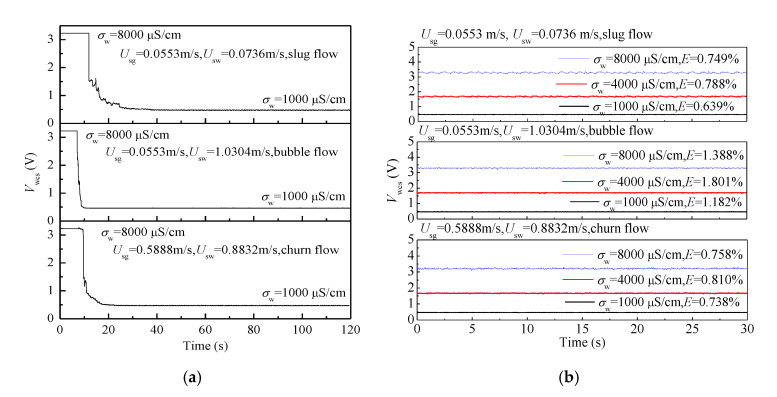
The performance of the water conductivity sensor to water conductivity measurement: (**a**) The sensor responses to the water conductivity change (from 8000 μS/cm to 1000 μS/cm) under different flow patterns; (**b**) The sensor outputs under different flow patterns and water conductivities.

The four channel outputs of the water holdup sensor to three typical flow patterns with different water conductivities are shown in Figure 6a–c. As seen, the output signals of four channels present slight differences due to the non-uniform phase distribution of two-phase flows. For slug flow, the output signals of liquid slugs present high voltage, and the output signals of Taylor bubbles present low voltage. For bubble flow, the output signals present high voltage with small and high frequency fluctuation, which are similar with the signals of liquid slug in slug flow. The signals of churn flow are similar to slug flow, but the frequency of fluctuation is high. The increase of the water conductivity increases the outputs of the sensor, and the resolution of the sensor to conductivity variation caused by water holdup is not affected. The performance of the water conductivity sensor to water conductivity measurement in gas-water two-phase flow is shown in Figure 7. Figure 7a presents the responses of the water conductivity sensor to the water conductivity changes (from 8000 μS/cm to 1000 μS/cm) under different flow patterns. As seen, the water conductivity sensor has good capability in tracking the water conductivity change under different flow patterns. The outputs of the sensor under different flow patterns and water conductivities are shown in Figure 7b. It can be seen that water conductivity sensor has stable outputs and a small relative error *E* of conductivity measurement.

Since the outputs of the water holdup sensor depend on the water holdup and water conductivity, the normalized conductivity $G_{e}^{*}$ is defined to eliminate the influence of water conductivity on water holdup extraction:

(12) $$G_{e}^{i}=\sigma_{m}^{i}/\sigma_{w}^{}\;(i=A,B,C,D)$$

where $\sigma_{m}^{i}$ presents the measured mixture conductivity from the *i*th channel (*i* = A, B, C, D). $\sigma_{w}^{}$ is the measured water conductivity. Then, the average of the four-channel normalized conductivities is:

(13) $$G_{e}^{*}=(G_{e}^{A}+G_{e}^{B}+G_{e}^{C}+G_{e}^{D})/4$$

**Figure 8:**
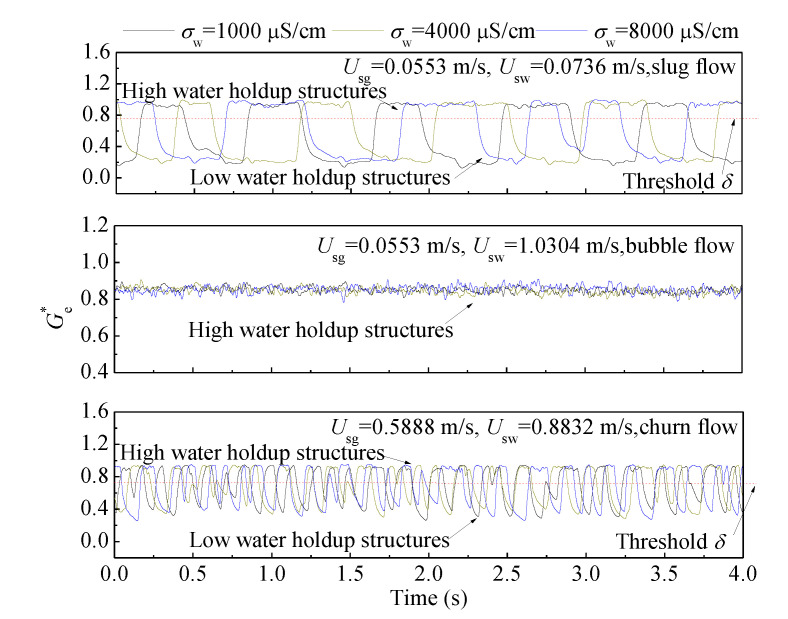
The $G_{e}^{*}$ of the water holdup sensor under different flow patterns and water conductivities.

As the water conductivity can be obtained, the $G_{e}^{*}$ of three typical flow patterns can be calculated using Equations (12) and (13), and the fluctuations of $G_{e}^{*}$ under different water conductivities are shown in Figure 8. It can be seen that $G_{e}^{*}$ is not affected by the change of water conductivity, and it only reflects the fluctuation of the water holdup. To deduce the water holdup from $G_{e}^{*}$, valid models need to be established. As seen in Figure 5, different flow patterns have different flow structures. For bubble flow, only bubbles with a small size are randomly distributed in the liquid phase, and these high water holdup structures are effectively reflected by $G_{e}^{*}$ in Figure 8. For slug flow and churn flow, the high-level parts of signals correspond to the high water holdup structures, which are liquid slugs in slug and churn flows. These flow structures are similar to bubble flow. The low-level parts of signals correspond to the low water holdup structures, which are Taylor bubbles in slug and large gas structures in churn flows. Since the low water holdup structures and the high water holdup structures are very different, the water holdup measurement models for each of them will be different. Therefore, accurate classification of different flow structures, obtaining the signals of different flow structures through a suitable threshold, and establishing the valid measurement models are the keys to accurately obtaining water holdup.

### 5.2. New Water Holdup Measurement Model

In terms of the water holdup measurement model, the Maxwell equation [29] assumes that non-interacting spherical particles with a uniform diameter whose conductivity is $\sigma_{d}$ are randomly distributed in conducting continuous media with a conductivity of $\sigma_{c}$. Therefore, it is valid for spherical particles with a uniform size at a low-volume fraction. Supposing the conductivity of mixture is $\sigma_{m}$, and the holdup of the dispersed phase is *Y*_d_, thus:

(14) $$\frac{\sigma_{m}-\sigma_{c}}{\sigma_{m}+2\sigma_{c}}=Y_{d}\frac{\sigma_{d}-\sigma_{c}}{\sigma_{d}+2\sigma_{c}}$$

For the gas-water flows, the conductivity of the dispersed gas phase $\sigma_{d}\;=\;0$, and the conductivity of the continuous water phase is $\sigma_{w}$. Considering the water holdup $Y_{w}\;=\;1\;-Y_{d}$, then, the water holdup model based on the Maxwell equation is:

(15) $$Y_{w}=\frac{3}{1+2/(\sigma_{m}/\sigma_{w})}\;$$

As the high water holdup structures, including bubble flow and the liquid slugs of slug and churn flows, conform to the above assumptions, the water holdup can be calculated using Equation (15). However, the low water holdup structures with large gas structures in slug and churn flows are different with the Maxwell assumption; therefore, Equation (15) might not be suitable for these structures in slug and churn flows.

Bruggemann [30] carried out work on the case of spheres with a broad range sizes and random distribution. His equation should be valid for spherical particles with a broad range of sizes at any volume fraction. The Bruggemann equation is:

(16) $$1-Y_{d}\;=\;\frac{\sigma_{m}/\sigma_{c}-\sigma_{d}/\sigma_{m}}{(\sigma_{m}/\sigma_{c}{)}^{1/3}(1-\sigma_{d}/\sigma_{m})}$$

For the gas-water flows, the water holdup model based on the Bruggemann equation is:

(17) $$Y_{w}={\left(\sigma_{m}/\sigma_{w}\right)}^{2/3}$$

where *Y*_w_, $\sigma_{m}$, and $\sigma_{w}$ are the water holdup, the conductivity of the mixture, and the water conductivity, respectively.

**Figure 9:**
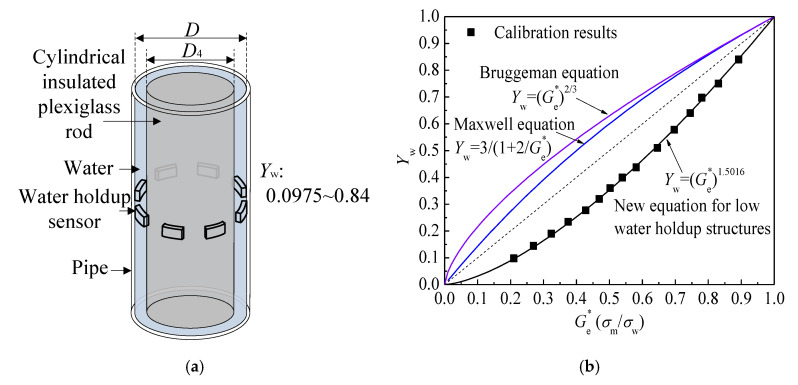
Establishment of the water holdup measurement model for low water holdup structures: (**a**) Calibration device; (**b**) Model establishment.

In order to evaluate the performance of the Maxwell equation and Bruggemann equation in water holdup measurement of low water holdup flow structures (Taylor bubbles of slug flow and large gas structures of churn flow), and to establish the valid water holdup measurement equation for these flow structures, we drew the curves of Equations (15) and (17) in Figure 9b. For the Taylor bubbles of slug flow and the large gas structures of churn flow, the shape of gas phase is similar to cylinder, and the conducting water exists between the gas structures and pipe wall. We simulated the gas structures with a cylindrical insulated plexiglass rod with a diameter *D*_4_ as shown in Figure 9a. The insulated plexiglass rod was placed in the center of the pipe, and the annular space between the insulated plexiglass rod and inner pipe wall was filled with water. By changing the diameter of the plexiglass rod, the $G_{e}^{*}$ of the sensor at different water holdup was obtained. Afterward, we present the water holdup under corresponding $G_{e}^{*}$ in Figure 9b. As seen, the Maxwell equation and Bruggemann equation are not suitable for measuring water holdup in low water holdup structures because the structures do not satisfy the assumption of the Maxwell equation and Bruggemann equation.

The theoretical model for calculating the water holdup of large gas structures in slug flow and churn flow is relatively lacking. For non-spherical dispersed phases, when researchers investigated the conducting properties of water-saturated rocks, they found that there is a power law relationship between normalized conductivity and porosity, and the power exponent is related to the geometry and distribution of dispersed phase [31,32]. De la Rue and Tobias [33] also measured the conductivities of random suspensions of spheres, cylinders, and sand particles in aqueous solutions according to the expressions in power law form. In this study, we established a new equation for the Taylor bubbles of slug and large gas structures of churn flow by the fitting method in the power law form in Figure 9b, and it can be expressed as:

(18) $$Y_{w}={\left(G_{e}^{*}\right)}^{n}\;n=1.5016$$

Finally, a new model for measuring water holdup in gas-liquid two-phase flow was established as shown in Equation (19). In practical measurement, the Maxwell equation is directly used to obtain the water holdup of bubble flow. For slug and churn flows, the $G_{e}^{*}$ of slug flow and churn flow shown in Figure 8 can be divided into signals of high water holdup structures and low water holdup structures by the threshold δ. For the high water holdup structures, the Maxwell equation is used to acquire the water holdup. For the low water holdup structures, the new equation established in Figure 9b is used to calculate the water holdup. Then, the weighted mean value of the water holdup as shown in Equation (19) is the final water holdup. The value of *a* can be determined as: *a* = *N*_h_/*N*, where *N*_h_ is the sampling points of signals of high water holdup structures, and *N* is the whole sampling points. The value of *b* can be determined as *b* = *N*_l_/*N*, where *N*_l_ is the sampling points of signals of low water holdup structures. The value of δ can be determined by comparing the measurement results with the real water holdup obtained by QCV:

(19) $$Y_{w}=\left\{ \begin{array}{ll} \frac{3}{1+(2/G_{e}^{*})} & \mathrm{bubble}\;\mathrm{flow}\\ a\frac{3}{1+(2/G_{e}^{*})}+b(G_{e}^{*}{)}^{1.5016} & \mathrm{slug}\;\mathrm{flow}\;\mathrm{and}\;\mathrm{churn}\;\mathrm{flow} \end{array}\right.$$

**Figure 10:**
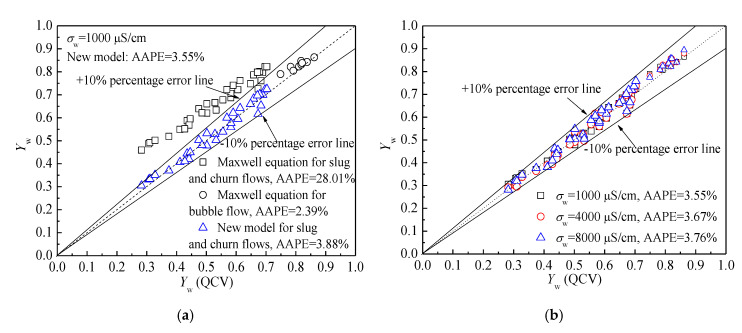
The calculation results using the new model: (**a**) Result comparison using the Maxwell equation and new model when the water conductivity is 1000 μS/cm; (**b**) Salinity independent water holdup measurement using the new model.

Figure 10a presents the calculation results using only the Maxwell equation and the new proposed model when the conductivity of water is 1000 μS/cm. It can be seen that the Maxwell equation has a good measurement accuracy for bubble flow, but for slug flow and churn flow, the measurement error is very big because of their different flow structures. The new model greatly improves the accuracy of water holdup measurement in slug flow and churn flow, and satisfactory results are achieved. The absolute average percentage error (AAPE) of water holdup measurement in three flow patterns using the new model is 3.55%.

The calculation results of water holdup using the new proposed method under different water conductivities are presented in Figure 10b. The water holdup sensor can acquire the water holdup with a high resolution under the conditions of different water conductivities, and the water conductivity is considered in the calculation process of $G_{e}^{*}$. Moreover, a new valid model is established for water holdup measurement for the three typical flow patterns. Hence, salinity independent water holdup measurement with high accuracy can be achieved, and the repeatability of the water holdup measurement with different water conductivities is satisfactory.

### 5.3. Salinity Independent Flow Velocity Measurement

**Figure 11:**
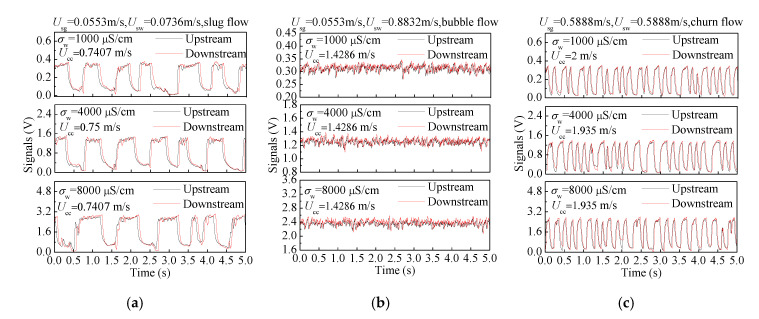
The signals of the velocity sensor and calculation results under three typical flow patterns and different water conductivities: (**a**) Slug flow; (**b**) Bubble flow; (**c**) Churn flow.

In this section, salinity independent mixture velocity and individual phase superficial velocity measurement are investigated. To obtain the mixture velocity using the cross-correlation method, the accurate measurement of cross-correlation velocity under the conditions of salinity change is important. The cross-correlation function in the cross-correlation method can be calculated using the following Equation (20):

(20) $$R_{\mathrm{xy}}(\tau )=\underset{T\to \infty }{\lim}\frac{1}{T}\int_{0}^{T}x(t)y(t+\tau )dt$$

where *x*(*t*) and *y*(*t*) present the upstream and downstream signals, respectively. *T* is the integral time, and *R*_xy_(*τ*) denotes the cross-correlation function. When *R*_xy_(*τ*) takes its maximum, the value of the time delay *τ* is the transit time *τ*_0_, then the cross-correlation velocity *U*_cc_ can be obtained by:

*U*_cc_ = *L*/*τ*_0_, (21)

where *L* represents the distance between the upstream sensor and downstream sensor. Figure 11 shows the outputs of the velocity sensor in three typical flow patterns with different water conductivities and the results of the cross-correlation calculation. It can be seen that the correlations between upstream signals and downstream signals are enhanced due to the increased fluid velocity caused by the center body. Due to the suitable conductivity detection method, the variation of water conductivity only changes the amplitude of the signal, but it does not affect the correlation between the upstream signal and downstream signal. Therefore, the cross-correlation velocities *U*_cc_ of the same flow conditions under different water conductivities are almost the same, and accurate measurement of *U*_cc_ can be guaranteed.

In order to deduce the mixture velocity and individual phase superficial velocity from the measured water holdup and cross-correlation velocity, valid flow velocity measurement models must be established. Because of the severe slippage effect and non-uniform phase distribution in gas-water flows, the establishment of models is challenging. For the mixture velocity measurement, the fact that the cross-correlation velocity corresponds to the kinematic wave velocity in the multiphase flow [34,35] has been found, and the mixture velocity measurement model can be reasonably established based on this theory [36,37]. Based on the work of Lucas and Jin [36], the relationship between cross-correlation velocity *U*_cc_ and mixture velocity in annular space *U*_m1_ in two-phase flows can be expressed as:

(22) $$U_{\mathrm{cc}}=C^{*}U_{m1}+B^{*}$$

The $C^{*}$ is given by:

(23) $$C_{}^{*}=C_{01}+Y_{g1}\frac{\partial C_{01}}{\partial Y_{g1}}$$

and $B^{*}$ is:

(24) $$B^{*}=U_{\infty }{\left(1-Y_{g1}\right)}^{n}[1-\frac{nY_{g1}}{1-Y_{g1}}]$$

where *C*_01_, *Y*_g1_, and *n* represent the distribution parameter, gas holdup, and droplet size exponent in the annular space, respectively. The $U_{\infty }$ is the terminal rise velocity of a single gas bubble relative to the continuous water phase.

**Figure 12:**
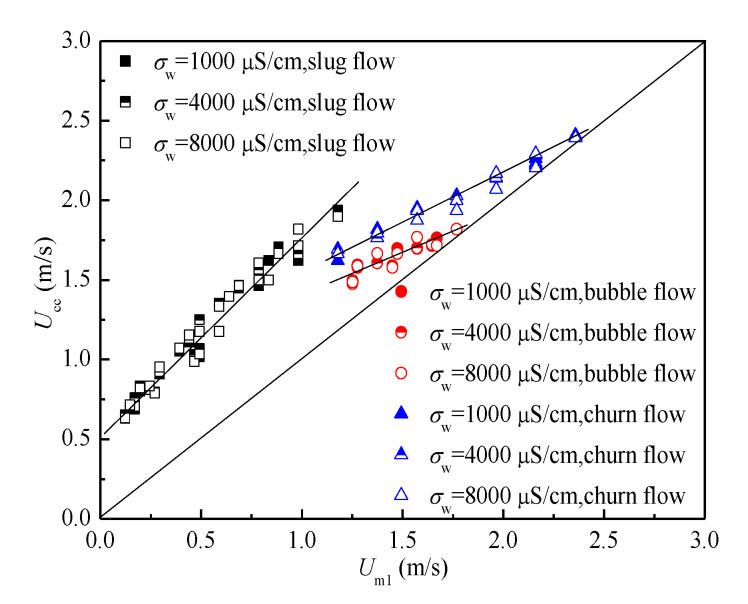
The relationships between *U*_cc_ and *U*_m1_ under three typical flow patterns and water conductivities.

We investigated the relationships between *U*_cc_ and *U*_m1_ in Figure 12. As seen, *U*_cc_ are different with *U*_m1_ because of the influence of $C^{*}$ and $B^{*}$. It is noteworthy that the *U*_cc_ has a nearly linear relationship with *U*_m1_ in the same flow pattern. Hence, the relationship between *U*_m1_ and *U*_cc_ is simplified because of the increased fluid velocity in the annular space. The change of water conductivity will not affect the calculation results of the cross-correlation velocity, so the relationships between the cross-correlation velocity and mixture velocity are similar under different water conductivity conditions. We used the relationship between *U*_cc_ and *U*_m1_ under the water conductivity of 1000 μS/cm to build the model by the fitting method in Figure 12. Therefore, salinity independent mixture velocity measurement can be achieved.

**Figure 13:**
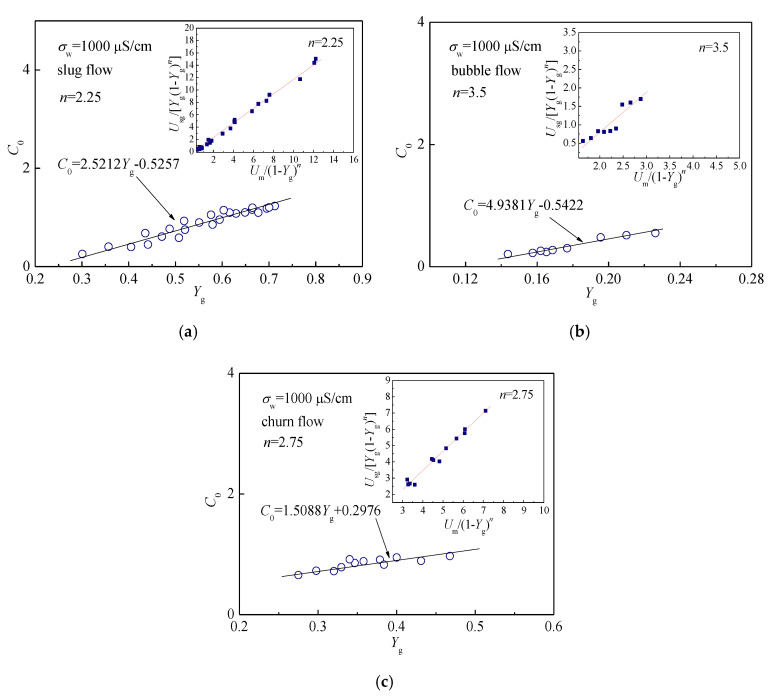
The determination of *n* and *C*_0_ in three typical flow patterns and water conductivity of 1000 μS/cm: (**a**) Slug flow; (**b**) Bubble flow; (**c**) Churn flow.

Considering the severe slippage effect and the non-uniform phase distribution in gas-water flows, the drift-flux model [38] was investigated and established for prediction of individual phase superficial velocity. The expression of drift-flux model is

(25) $$\frac{U_{\mathrm{sg}}}{Y_{g}}=C_{0}U_{m}+U_{\infty }{\left(1-Y_{g}\right)}^{n}$$

where *U*_sg_ is the gas phase superficial velocity. *Y*_g_, *U*_m_, *C*_0_, and *n* are the gas holdup, mixture velocity, distribution parameter, and droplet size exponent in the 20 mm inner diameter pipe, respectively. The $U_{\infty }$ can be obtained according to [39]. To obtain the values of *C*_0_ and *n*, we investigated the relationships between $U_{m}/{\left(1-Y_{g}\right)}^{n}$ and $U_{\mathrm{sg}}/Y_{g}{\left(1-Y_{g}\right)}^{n}$ of different flow patterns in Figure 13 through setting different values of *n* in Equation (25). Because of the realization of salinity independent mixture velocity and water holdup measurement, in the process of model establishment, *U*_m_ and *Y*_g_ are the measurement results under the water conductivity of 1000 μS/cm. For slug flow as shown in Figure 13a, when *n* = 2.25, the data points present a better linear relationship, and the value of *n* in slug flow is determined as 2.25. Using the same method, the values of *n* in bubble and churn flows are determined as 3.5 and 2.75, respectively. We regard that the values of *C*_0_ depend on *Y*_g_, and the relationship between *C*_0_ and *Y*_g_ under different flow patterns are shown in Figure 13. Then, we obtained the relationship between them using the fitting method. Finally, we substituted the values of *n* and the relationships between *C*_0_ and *Y*_g_ into Equation (25), and the expression of drift-flux model for gas-water two-phase flow was obtained.

The final expression of the mixture velocity and individual phase superficial velocity measurement models established under the water conductivity of 1000 μS/cm for gas-water two-phase flow can be expressed as:

(26) $$\left\{ \begin{array}{l} U_{m}=(U_{\mathrm{cc}}-0.5194)/1.6711\\ U_{\mathrm{sg}}=(2.5212Y_{g}-0.5257)Y_{g}U_{m}+U_{\infty }Y_{g}(1-Y_{g}{)}^{2.25} \end{array}\right.\;\mathrm{Slug}\;\mathrm{flow}$$

(27) $$\left\{ \begin{array}{l} U_{m}=(U_{\mathrm{cc}}-0.8994)/0.684\\ U_{\mathrm{sg}}=(4.9318Y_{g}-0.5422)Y_{g}U_{m}+U_{\infty }Y_{g}(1-Y_{g}{)}^{3.5} \end{array}\right.\;\mathrm{Bubble}\;\mathrm{flow}$$

(28) $$\left\{ \begin{array}{l} U_{m}=(U_{\mathrm{cc}}-0.9297)/0.8124\\ U_{\mathrm{sg}}=(1.5088Y_{g}+0.2976)Y_{g}U_{m}+U_{\infty }Y_{g}(1-Y_{g}{)}^{2.75} \end{array}\right.\;\mathrm{Churn}\;\mathrm{flow}$$

**Figure 14:**
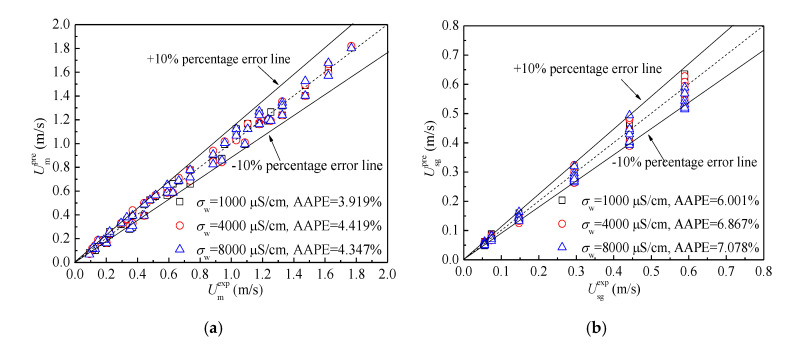
Salinity independent flow velocity measurement: (**a**) Mixture velocity; (**b**) Gas phase superficial velocity.

Then, we used the established models under the water conductivity of 1000 μS/cm to predict the mixture velocity $U_{m}^{\mathrm{pre}}$ and gas phase superficial velocity $U_{\mathrm{sg}}^{\mathrm{pre}}$ under different water conductivities. The results are shown in Figure 14, and the prediction accuracy of flow velocities is satisfactory. Therefore, salinity independent mixture velocity and individual phase superficial velocity measurement can be realized. The results of three measurements with different water conductivities are all satisfactory, which reflect the good repeatability of the velocities’ measurement.

## 6. Conclusions

In this study, a methodology for flow measurement using conductance method in gas-liquid two-phase flow with salinity change was proposed. The methodology includes the suitable conductivity detection method, the strategy of using combined sensors, and the measurement models of flow parameters. Based on the proposed methodology, we designed the combined conductance sensor system, and investigated the water holdup measurement model and flow velocity measurement models under the conditions of water conductivity change for the first time. The conclusions can be summarized as follows:

1. The linear relationship between the sensor output and water conductivity is more suitable for conductivity detection under the conditions of changing water conductivity. Using the water holdup sensor, the velocity sensor, and water conductivity to form a combined conductance sensor system is an effective strategy to achieve salinity independent flow measurement in gas-water flows. As the sensor output is positively linearly proportional to the conductivity, the water holdup sensor and velocity sensor can capture the conductivity variation caused by water holdup under the conditions of water conductivity change with high and constant resolution. The water conductivity sensor can dynamically obtain water conductivity in the gas-liquid two-phase flow. In the calculation of water holdup, water conductivity is considered, thus the influence of salinity can be eliminated.
2. For the water holdup measurement, a new water holdup measurement model based on flow structures was proposed. The bubble flow and liquid slugs in slug flow and churn flow were classified into high water holdup flow structures, and Taylor bubble of slug flow and the large gas structures of churn flow were classified into low water holdup flow structures. For the high water holdup flow structures, the Maxwell equation can achieve satisfactory water holdup measurement results. For the low water holdup flow structures, the Maxwell equation and Bruggemann equation all have limitations. Based on the characteristics of these structures, a new equation was established. Finally, a new water holdup measurement model was established to achieve salinity independent water holdup measurement in gas-water flows.
3. As the output of the velocity sensor is linearly proportional to the conductivity, so the change of water conductivity only affects the amplitude of signals, and the resolution of the sensor to the conductivity variation caused by water holdup is not affected. Moreover, the fluid velocity is increased in the annular space, which enhances the correlation of upstream signal and downstream signal and simplifies the relationship between mixture velocity and cross-correlation velocity. The drift-flux model that considers the droplet size exponent, distribution parameter, and slippage velocity into consideration was established, and salinity independent flow velocity measurement was achieved satisfactorily. This paper presents a methodology to realize salinity independent flow measurement in gas-liquid flows using the conductance method from the perspective of theoretical analysis and experimental verification for the first time. It contributes to the application of the conductance method in dynamically monitoring oil wells with water salinity change. It is worth noting that for the conductance method, the change of temperature also affects the water conductivity; therefore, the method discussed in this study can also be applied to flow conditions with a changing temperature.

## Nomenclature

*a*	Proportion of high water holdup structures	*U*_sg_	Gas superficial velocity
*b*	Proportion of low water holdup structures	*U*_sw_	Water superficial velocity
*C*_0_	Distribution parameter	$U_{\infty }$	Terminal rise velocity of a single gas bubble relative to the continuous water phase
*C*_01_	Distribution parameter in annular space	*V*_s_	Value of exciting voltage signal
*D*_1_	Diameter of insulated flow deflector 1	*V*_1_	Voltage output 1
*d*_1_	Distance between two ring-shape electrodes	*V*_out_	Sensor output
*D*_4_	Diameter of cylindrical insulated plexiglass rod	*V* ^A^	Voltage output of channel A
*E*	Relative error	*V* ^B^	Voltage output of channel B
*G*	Value of equivalent conductance	*V* ^C^	Voltage output of channel C
$G_{e}^{*}$	Normalized conductivity	*V* ^D^	Voltage output of channel D
*H*	Axial height of electrodes	*V*_wcs_	Output of water conductivity sensor
*h*	Height of electrode	*V* ^up^	Output of upstream sensor
*H*_1_	Length of insulated flow deflector 1	*V* ^down^	Output of downstream sensor
*H*_4_	Distance between insulated flow deflector 1 and insulated flow deflector 2	*Y*_d_	Holdup of dispersed phase
*I*	Value of current	*Y*_g_	Gas holdup
*I*_s_	Value of exciting current signal	*Y*_g1_	Gas holdup in annular space
*k*	A constant relating to the configuration of electrode	*Y*_w_	Water holdup
*L*	Distance between upstream and downstream sensors	*θ*	Field angle of electrodes
*L*_1_	Distance between upstream sensor and the head of insulated flow deflector 1	δ	Threshold
*N*	Amplification factor	σ	Conductivity
*n*	Droplet size exponent	$\sigma_{c}$	Conductivity of conducting continuous media
*R*_m_	Value of equivalent resistance	$\sigma_{d}$	Conductivity of dispersed phase
*R*_ref_	Value of reference resistance	$\sigma_{m}$	Conductivity of mixture
*R*_1_	Resistance 1	$\sigma_{w}$	Water conductivity
*R*_2_	Resistance 2	$\sigma^{A}$	Conductivity measured by channel A
*R*_3_	Resistance 3	$\sigma^{B}$	Conductivity measured by channel B
*R*_4_	Resistance 4	$\sigma^{C}$	Conductivity measured by channel C
*R*_5_	Resistance 5	$\sigma^{D}$	Conductivity measured by channel D
*R*_6_	Resistance 6	$\sigma^{\mathrm{up}}$	Conductivity measured by upstream sensor
*T*	Radial thickness of electrodes	$\sigma^{\mathrm{down}}$	Conductivity measured by downstream sensor
*U*_cc_	Cross-correlation velocity		
*U*_m_	Mixture velocity		
*U*_m1_	Mixture velocity in annular space		
